# Mind the matter: Active matter, soft robotics, and the making of bio-inspired artificial intelligence

**DOI:** 10.3389/fnbot.2022.880724

**Published:** 2022-12-15

**Authors:** David Harrison, Wiktor Rorot, Urte Laukaityte

**Affiliations:** ^1^Department of History and Philosophy of Science, University of Cambridge, Cambridge, United Kingdom; ^2^Leverhulme Centre for the Future of Intelligence, Cambridge, United Kingdom; ^3^Konrad Lorenz Institute for Evolution and Cognition Research, Vienna, Austria; ^4^Human Interactivity and Language Lab, Faculty of Psychology, University of Warsaw, Warsaw, Poland; ^5^Department of Philosophy, University of California, Berkeley, Berkeley, CA, United States

**Keywords:** multiple realisability, fine-grained functionalism, functionalism, soft robotics, active matter physics, basal cognition, artificial intelligence, embodied cognition

## Abstract

Philosophical and theoretical debates on the multiple realisability of the cognitive have historically influenced discussions of the possible systems capable of instantiating complex functions like memory, learning, goal-directedness, and decision-making. These debates have had the corollary of undermining, if not altogether neglecting, the materiality and corporeality of cognition—treating material, living processes as “hardware” problems that can be abstracted out and, in principle, implemented in a variety of materials—in particular on digital computers and in the form of state-of-the-art neural networks. In sum, the matter *in se* has been taken not to matter for cognition. However, in this paper, we argue that the materiality of cognition—and the living, self-organizing processes that it enables—requires a more detailed assessment when understanding the nature of cognition and recreating it in the field of embodied robotics. Or, in slogan form, that the matter matters for cognitive form and function. We pull from the fields of Active Matter Physics, Soft Robotics, and Basal Cognition literature to suggest that the imbrication between material and cognitive processes is closer than standard accounts of multiple realisability suggest. In light of this, we propose upgrading the notion of multiple realisability from the standard version—what we call 1.0—to a more nuanced conception 2.0 to better reflect the recent empirical advancements, while at the same time averting many of the problems that have been raised for it. These fields are actively reshaping the terrain in which we understand materiality and how it enables, mediates, and constrains cognition. We propose that taking the *materiality* of our embodied, precarious nature seriously furnishes an important research avenue for the development of embodied robots that autonomously value, engage, and interact with the environment in a goal-directed manner, in response to existential needs of survival, persistence, and, ultimately, reproduction. Thus, we argue that by placing further emphasis on the soft, active, and plastic nature of the materials that constitute cognitive embodiment, we can move further in the direction of autonomous embodied robots and Artificial Intelligence.

## Introduction

Standard approaches to understanding cognition—and the wider goal of recapitulating it on simulated platforms or in the field of robotics—have tended to neglect the importance of the materiality of the body and its relevance for constraining, enabling, and mediating cognition. This contention is centred right at the origin of the cognitive sciences and is typically framed in terms of *multiple realisability*. It is often argued, then, that cognition is a species of software that, in principle, is instantiable in any back-of-the-envelope set of materials so long as they are “suitably organized” (Putnam, 1975). As Hilary Putnam once put it, “We could be made of Swiss cheese and it wouldn't matter” (Putnam, 1975: 291). Although few authors would defend this version of multiple realisability (MR) today (see Polger and Shapiro, 2016 for a state-of-the-art discussion of the philosophical literature), the belief that the materiality of cognition is mostly a “hardware” problem with the truly interesting explanandum being cognitive “software” that sits above still permeates much of the theoretical and philosophical literature. However, as we will see below, much turns on what it means to be “suitably organized” and it is by no means clear that any pell-mell set of materials could instantiate the complex dynamics on which cognition depends.

Thus, by making recourse to recent experimental, material, and theoretical developments in active matter physics and soft robotics, in this paper we argue that the separation of non-mental, living “hardware” and cognitive “software” has grown increasingly suspect—and that a harder pivot toward the materiality of the body and cognition is now needed. In other words, what we are claiming is not (or not simply) that the body matters to cognition (a view that tacitly supposes the body in service of a more or less higher-order, more or less unified, cognitive subject), but rather that the body itself—at varying levels of organization—exhibits cognitive capacities through and through: from cellular activities entrained to regulating morphology, development, and intercellular communication; to tissue complexes and system functioning; through to more baroque appearances of cognitive sophistication encapsulated in cephalopod, arthropod, avian, and mammalian brains—Darwin's “endless forms most beautiful” (*Origin of Species*). In a slogan expressed elsewhere (Levin, 2019, 2020; Levin and Dennett, 2020), this is cognition *all the way down*, not just proprietary to a unified subject. Making sense of the theoretical commitment behind this claim and how it contributes to the development of intelligent machines is the main goal of our paper. It is thus worth clarifying at the outset that our discussion of robotics pertains to what we could consider Autonomous Robots (AR), i.e., autonomous embodied systems capable of recursive self-organization, goal-directedness, and agency—the ability to flexibly and actively select goals relative to its “existential needs” (Froese, 2016; Egbert, 2022) and remain the kind of system it is (Man and Damasio, 2019). The key here, as we see it, is to understand how the matter matters to being this kind of system.

The picture we would like to work against is one of neurocentrism that cleaves neuronal (and cognitive) activity from the living, developmental, and morphogenetic processes for which nervous systems originally evolved (see Lyon, 2006; Van Duijn et al., 2006; Keijzer et al., 2013; Newman, 2016, 2019, 2022; Levin, 2019, 2020; Fields and Levin, 2020; Sims, 2020, 2021; Fields et al., 2021; Jekely, 2021; Lyon et al., 2021; Wan and Jekely, 2021). It is this sense in which we think the tacit commitments of MR—the in principle cleaving of active, living processes and cognitive ones—deserve a reconsideration. As Peter Godfrey-Smith remarks, philosophers and cognitive scientists tend to operate with a “picture in which living activity is a kind of non-mental substrate, and then evolution lays a computer—the nervous system—on top of the merely living, after which cognition and subjective experience result” (Godfrey-Smith, 2016a: 496). This can be seen in the very structure of the cognitive sciences and its lack of (explicit) emphasis on the life sciences. That is, while biological perspectives have influenced theorising about the mind [e.g., autopoiesis (Varela et al., 1993; Weber and Varela, 2002) and enactivism (Di Paolo et al., 2017)], they have not furnished real competitive alternatives to more mainstream cognitivism and computationalism [see Meyer and Brancazio (2021) for an insightful discussion]. To this day, it is common to see neurons and the brain—the “stuff” of cognition—almost wholly abstracted from the life processes in which they are embedded.

Here, we hope to cast doubt on the (un)happy divorce between material and cognitive processes by suggesting that looking toward recent developments in soft robotics (Man and Damasio, 2019; Blackiston et al., 2021; Bongard and Levin, 2021; Kaspar et al., 2021; Kriegman et al., 2021), active matter physics (Hanczyc and Ikegami, 2010; Needleman and Dogic, 2017; McGivern, 2020; Egbert, 2021), and basal cognition research (Lyon, 2006, 2015; Van Duijn et al., 2006; Newman, 2016, 2019, 2020, 2021; Levin, 2019, 2020; Bechtel and Bich, 2021; Lyon et al., 2021) complicates any cleaving of cognition from its living, material context. In light of recent empirical advancements, we argue now is a good time to revisit our philosophical assumptions regarding the MR of the cognitive and suggest that a more promising path in the development of AR and Artificial Intelligence (AI) is to take the materiality of cognition *more*, not less, seriously—a position explicitly disallowed in standard philosophical positions on MR. Our argument thus consists of two interlocked moves: first, we identify a set of assumptions that structure the debate on MR and that generate strong intuitions regarding the mental-physical interaction that have historically discouraged taking the materiality of cognition seriously. Second, we propose a path to AR that explores a more thoroughgoing, “radically embodied” approach: one that does not see the body as a “non-mental” substrate on top of which cognitive software (the nervous system) is placed, but instead depicts cognition as a more fundamental feature of cellular (read: living) activity and self-organizing processes in far-from-equilibrium conditions that are then *scaled up* in appropriate ways to arrive at more sophisticated multicellular animals.

At this point, it is worth being explicit about three things. First, we draw a strong connection between living and cognitive processes—consistent with much of the literature on the so-called life-mind continuity thesis (Maturana and Varela, 1980; Thompson, 2010; Sims, 2021). *Prima facie*, this would seem to undermine our goal of constructing AR, as it would suggest some of the prototypical cognitive behaviours we see in certain soft-bodied robots and active material systems (examined in Section Active matter and soft robotics: Novel approaches to cognition and embodied robotics) cannot qualify as such due to their non-living nature. We believe this problem can be ameliorated, however, by adopting a conception of cognition that depicts the living and developmental side of the process as a more general feature of self-organizing systems in far-from-equilibrium thermodynamic states that must act in a denumerable set of ways to remain the kind of system it is. Simply put: we accept here a view of “life” which does not presuppose particular material foundations (e.g., carbon-based), but rather takes it to be an organizational feature (cf. Moreno and Mossio, 2015). Under this view, then, cognition can be seen as tailored for the homeostatic processes that underpin goal-directed, autonomous, and agentic behaviour (see Pezzulo et al., 2015), and a non-living system would fall closer to the cognitive the more it embodies such dynamics. This brings us to the second consideration, namely, that the lynchpin for our discussion of cognition turns around the notion of “existential needs” and can be explicated, following Lyon et al. (2021), in relation to the set of sensory and information processing mechanisms organisms have for familiarising themselves, valuing, and interacting actively with the environment in order to meet the existential needs of survival, persistence, growth, and reproduction. In the literature, this is often called *basal* cognition, as it refers to a set of mechanisms and capacities with highly ancient, highly distributed origins. We earmark this for now and return to it in Section How fine-grained functional details matter to cognition for a more nuanced discussion. Lastly, it is important to clarify the scope of the present paper. While we engage a diverse range of empirical literature—from active matter physics to soft robotics—we ultimately position the paper at a theoretical level that targets the metatheoretical assumptions that scaffold debates on cognition and mind in Robotics and AI. Stated differently, what we are trying to target is a certain set of assumptions and presuppositions that have historically dominated this field, which complicate taking the materiality of embodiment further than is currently being explored in emerging areas of the life and mind sciences. However, while the present piece is considered theoretical, we believe it encourages actionable and implementable possibilities for creating autonomous systems by incorporating elements of self-organizing dynamical systems (Pfeifer et al., 2007)—as is increasingly explored within the domain of active matter physics and soft robotics.

Our focus on existential needs depends on recent research advocating for taking the materiality of our embodiment further than mainstream embodied cognition has commonly done (cf. Müller and Hoffman, 2017). We thus place a premium on the very processes, goals, and demands of a living body that are normally elided from more theoretical meditations on the cognitive. A similar approach has been proposed by Man and Damasio (2019) who suggest we transition away from the hard parts that typify traditional roboticist approaches to fragile, vulnerable, and soft materials characteristic of organismic embodiment. The fundamental innovation introduces homeostasis and risk-to-self as the warp and weft of cognitive embodiment: “These machines [our AR] have physical constructions—bodies—that must be maintained within a narrow range of viability states… Rather than up-armouring or adding raw processing power to achieve resilience, we begin the design of these robots by, paradoxically, introducing vulnerability” (Man and Damasio, 2019: 449). Indeed, similar to Man and Damasio, we believe a shift from embodied (*simpliciter*) AI to homeostatic and precarity driven AI is the key requirement for the coming generations of AR. This puts more emphasis on the material *processes* and material situation than simply focusing on embodiment full stop. The second notion we depend on has already been mentioned: that of precarity. Tom Froese has argued that the nature of our embodied precariousness (risk-to-self) is essential for agency and the problem of meaning [we might call this a species of the frame problem (McCarthy and Hayes, 1969): why would an artificial agent come to care about its existence and actions on which it depends?]. He writes, “The precariousness that is intrinsic to all organismic, and therefore also of all mental, existence is the original reason why things matter to that individual being” (Froese, 2016: 34). That is, organisms are cognitive agents with meaningful engagements with the world because, and not in spite of, their fundamentally precarious nature. Importantly, this can be also expressed in terms of values and value-realising, which some believe to be the main force driving and organizing action in cognitive agents (cf. Hodges and Baron, 1992; Hodges and Raczaszek-Leonardi, 2021). Precarity is the minimal form of valence, hence enabling cognition and agency (cf. Lyon and Kuchling, 2021). Thus, if “the problem with AI”, as John Haugeland famously put it, “is that it doesn't give a damn” (Haugeland, 1998), then we explore how an active matter lens focusing on specific material reconfigurations that enable systems to maintain themselves in far-from-equilibrium conditions can make headway on this most defining of problems for computer science: autonomous robots that might one day give a damn.

The structure of this paper will be as follows. In Section Traditional vs. fine-grained functionalism we briefly overview some of the theoretical and philosophical literature on MR before suggesting that the tenability of its more radical iterations turns on a few key (and, as we would like to suggest, misguided) assumptions that are in need of a rethink in light of recent theoretical and empirical advancements. Instead, we aim to put forward a version of MR that *both* takes the materiality of cognition seriously *and* allows for cognition to be instantiable in alternative media. Section Active matter and soft robotics: Novel approaches to cognition and embodied robotics turns toward the state-of-the-art research to suggest that the domains of active matter physics and soft robotics encourage us to reformulate how we understand the mental-physical interaction. Finally, in Section How fine-grained functional details matter to cognition we defend a “cognition all the way down” approach to the development of bio-inspired AI.

## Traditional vs. fine-grained functionalism

Before putting forward our positive proposal, we need to highlight some of the deeply entrenched philosophical assumptions of the current programme of artificial intelligence that we believe to be detrimental.

The methodology of contemporary AI research is built on top of the philosophical programme of functionalism in philosophy of mind. Functionalism was developed in the second half of the 20th century in response to the issues surrounding the physicalist mind-brain identity theories dominant at the time. In the late 1960s Hilary Putnam advanced a novel line of thought, which sought to establish that mental states (and properties) are *functional* states. From the onset of this view functions, understood as causal mappings between sensory inputs, other internal states, and behavioural outputs (Levin, 2021), were defined in broadly computational terms. This allowed philosophers to disentangle cognition from its neurophysiological, material basis and argue that psychological (which was the main term used for what we call “cognition”) processes are “General” (see Polger and Shapiro, 2016: 15), i.e., shared across species, and in fact that psychological functions can be realised by entirely distinct types of systems—not only differently organized animal brains but also a variety of non-biological systems. A special case of interest concerned digital computers, which seem under this view to be well suited for realising psychological processes. This is the idea that has come to be known as the claim of “multiple realisability” (MR) of psychological states.

As Chirimuuta (2018) observes, functionalist theory of mind and the concept of multiple realisability hold a unique status in philosophy as views to which a “near majority of philosophers have subscribed to, and for more than one generation”. However, in an important book, Polger and Shapiro (2016) argue that the view of multiple realisability is not, in fact, borne out by the empirical evidence accrued over time. The main thrust of Polger and Shapiro's arguments is aimed at the tenuous distinction between inherent superficial variation in the biological world and deeper differences which are in fact responsible for the multiple realisation of cognitive functions. This in fact turns out to be damning regardless of whether one assumes that mental states are multiply realisable functions (the ontological, objective stance) or whether one argues that they can be explained as multiply realisable functions (the epistemological, subjective view).^1^ Their points are targetted at what can be called “MR 1.0” (Chirimuuta, 2018) and, in result, call for a rejection of traditional functionalism. MR 1.0 is, Polger and Shapiro argue, untenable given contemporary empirical evidence.

The functionalist account has suffered from other important theoretical criticisms as well, among which we may highlight the dual objection that functionalism is either (1) too liberal under one reading or else (2) too chauvinistic under another; and, what is more, there are no other interpretations available to it. Following the first option, under which functionalism fails to specify any restriction on the domain of physical systems, it will assign mindedness to entities that should not be viewed as minded. In fact, an important argument in this vein comes from Putnam himself, who later in his life rejected the computational theory of mind. Putnam (1988) proves the theorem that “[e]very ordinary open system is a realisation of every abstract finite automaton”, which would lead to an uncontrolled expansion of systems that we should consider as realising cognitive functions—contrary to our experience with the world. The opposite argument has been initially suggested by Ned Block. Block (1978) argues that any version of functionalism that avoids liberalism by opting for some set of physical specifications falls into biological chauvinism and hence denies mentality to creatures that we would ordinarily consider as such. His reason for the claim is the thought that one could always conceive of some system that would fail to meet the physical constraints and yet intuitively seem to possess psychological states.

However, despite the problems with traditional functionalism, the conviction that multiple realisability is an important feature of the cognitive remains widespread among researchers. In fact, it plays a significant role not only in the study of cognition but in the life sciences at large. This led Chirimuuta to propose that instead of rejecting MR altogether, we need to carefully update this notion to account for the role that functional thinking beyond traditional functionalism plays in biology.

Chirimuuta's conception of “MR 2.0” is grounded in observations of how the consideration that certain biological processes are best described as functions that can in principle be multiply realised is an important assumption that allows scientists to make decisions with regard to what physical properties can be safely ignored in their experiments, reducing the complexity of the problem to be studied. This conception of MR, or so Chirimuuta suggests, allows us to maintain that “[i]t can both be true that the material from which the nervous system is built (i.e., living, metabolizing cells) is crucial to their function *and* that those functions are multiply realised” (Chirimuuta, 2018: 411). In particular, this view lets us appreciate that the Heraclitean nature of biological material—its ability to preserve integrity through “continual turnover of matter and energy” (Chirimuuta, 2018: 411)—is crucial for understanding the functioning of cognition [as Godfrey-Smith (2016a) also argues], but at the same time a roadblock to the success of purely reductive methodologies, as this constant shifting obfuscates functionally relevant patterns which occur at a meso-level of description.

This updated view of MR is, in fact, compatible with the idea of “fine-grained functionalism” advanced by Godfrey-Smith (2016a). While Godfrey-Smith explicitly rejects the idea of MR, his arguments are aimed at the concept of MR 1.0. The traditional functionalist account, which builds on the older concept of MR, can be characterised, in Godfrey-Smith's terms, as “coarse-grained functionalism.” The two are distinguished by the level of organization that they focus on in identifying and characterising the relevant states and processes. Godfrey-Smith accepts a multi-layered view of reality and concedes that “[t]here are reasonable coarse-grained senses of ‘learn’ and ‘perceive’ in which anything with the right coarse-grained functional profile, including a robot, does learn and perceive” (Godfrey-Smith, 2016a: 501). However, he moves on to argue, the systems that we know to be cognitive and proto-cognitive, i.e., a variety of organisms, have an entirely different fine-grained make-up. Not only is it important that in living systems “the information processing side of its activity is integrated with the metabolic side” (Godfrey-Smith, 2016a: 502) but the small spatio-temporal scale at which cellular metabolism occurs has several unique characteristics. In particular, the cells are full of a *molecular storm* with “unending spontaneous motion [...]. Larger molecules rearrange themselves spontaneously and vibrate, and everything is bombarded by water molecules, with any larger molecule being hit by a water molecule trillions of times per second.” The ubiquitous electrical charge is just one form of energy present, as chemical, kinetic, and electrostatic energy are constantly transduced into one another. Each part of the cell is subject to forces stronger than it can exert and causality is best perceived as “biasing tendencies in the storm, nudging random walks in useful directions” (Godfrey-Smith, 2016a: 485–487). Cellular metabolism arises from this material volatility and constant flux and, as Godfrey-Smith underscores, principles governing it remain crucial for the processes that constitute cognition, due to their co-evolution.

While the exact dependence of the mind on these low-level processes remains an open question, Godfrey-Smith argues that fine-grained functionalism can account for the failure of traditional functionalist approaches to understanding and engineering minds. Consider a machine—a computer—or a cyborg; even if it has similar coarse-grained functions, it will be lacking the fine-grained functions which depend on the living (i.e., far from thermodynamic equilibrium) organization of biological organisms. It may be capable of “sensing” or “learning”, but these terms, or so Godfrey-Smith argues, are broad and coarse-grained, such that they do not rely on a similarity between the fine-grained functional profiles of sensing machines and sensing humans. Reality is multi-scaled and so focusing only on the scale of such coarse-grained properties will not yield the kind of understanding of cognitive processes we need to build intelligent artificial machines.

For Godfrey-Smith this view leads to a rejection of MR altogether but that is the case only for the traditional conception we call “MR 1.0”. “The finer-grained features are not merely ways of realising the cognitive profile of the system. They matter in ways that can independently be identified as cognitively important”, he argues (Godfrey-Smith, 2016a: 503). He indicates the inherent historicity of neurons—the change in their functional profile resulting from their own activity—as an example. This argument paves the way for Chirimuuta's upgraded notion of MR 2.0, which would hold that fine-grained functions and the material basis of cognition need to be centred in their own right, but could still, at least in principle, be multiply realised. Interestingly, a related point has in fact been a source of criticism for Godfrey-Smith's view raised by Brunet and Halina (2020), who discuss the existence of molecular machines—computers which preserve some of the low-level characteristics indicated by Godfrey-Smith—as an argument for the possibility of developing artificial sentience, which Godfrey-Smith appears to deny. However, given the discernible compatibility of Chirimuuta's MR 2.0 with Godfrey-Smith's fine-grained functionalism, it is more useful to consider Godfrey-Smith's rejection of contemporary approaches to AI to be concerned solely with their focus on coarse-grained functions.

To this list of grievances with regard to the traditional functionalist assumptions underpinning the current AI frameworks we may add one more, namely, that the coarse-grained functions they try to realise *in silico* are inherently highly complex. These are usually specific to a human way of engaging with the world, loaded with folk-psychological ideas, and disjointed from their evolutionary and developmental trajectory. In result, they are disconnected from the various scaffolds that biological intelligences use for the same purpose. This means that when trying to implement a particular psychological function in a computer AI researchers face a much more difficult problem than the one that evolution faces. If this consideration is correct, a “molecular computer” of the sort examined by Brunet and Halina (2020) would not be sufficient to be deemed a promising candidate for sentience, as developing the requisite coarse-grained functions on this platform would constitute a similarly difficult problem as in the case of silicon-based computing. This is because Brunet and Halina's view relies on the implicit distinction between the computational “hardware” of molecular computers and cognitive “software” (problematised in the introduction and discussed in greater detail in Section How fine-grained functional details matter to cognition). They are interested in the possibility of designing “universal Brownian circuitry capable of extracting useful computation from nano-scale fluctuations” (Brunet and Halina, 2020: 233) and instantiating cognitive processes on top of this circuitry. But that means that their understanding of the functions of cognition remains coarse-grained and hence disjointed from the properties of fine-grained functions. As a result, an AI researcher working on this platform would face a problem as difficult as when working on computing platforms employing standard, von Neumann architecture. The necessary missing step, consistent with fine-grained functionalism, seems to be the use of competent, intelligent parts in the manner suggested by work within basal cognition (e.g., Levin, 2019). We will explore this view in detail in Section How fine-grained functional details matter to cognition.

It is important to note that while both Godfrey-Smith and Chirimuuta leave their claims about the relevance of materiality for cognition at a pretty abstract and general level, we believe that several interconnected research fields—particularly active matter physics and soft robotics that are the focus of the current paper—allow for substantiating these claims further. Notably, doing so lets us draw some initial hypotheses about what “suitable organization” presumed by fine-grained functionalism could consist in and how metabolism may fit into this picture. We turn to the discussion of these disciplines in the next section.

## Active matter and soft robotics: Novel approaches to cognition and embodied robotics

In the previous section we overviewed some of the contemporary literature on MR, specifically regarding the cognitive. We referenced the fact that some of the basic pretenses of MR 1.0 seem to have grown increasingly suspect in light of empirical advancements in the cognitive and life sciences. Indeed, the crux—for our argument—is the condition that the material configurations instantiating cognition be “suitably organized”, a requirement that is a lot more stringent than proponents of MR 1.0 would allow. To this end, we began to suggest that recent developments in the areas of soft robotics and active matter physics hint that, while dimensions and aspects of cognitive systems can be manifested in alternative media, they do so *insofar* as they approximate the organizational, living, and developmental dynamics to organismic cognition—a position that we have called fine-grained functionalism.^2^ Thus, this section turns toward the empirical basis for something like fine-grained functionalism to adumbrate how the material out of which embodied agents are constituted is integral to sustaining the self-organizing dynamics on which cognition depends. Our main goal, then, is to suggest how a more thoroughgoing, “radically embodied” approach to AR and AI supplies the requisite tools to advance the field toward intelligent, plastic, and adaptive machines (Man and Damasio, 2019; Pishvar and Harne, 2020; Kaspar et al., 2021).

Before continuing, it is worth anticipating briefly why this approach is, or so we want to suggest, a more thoroughly embodied approach than previous iterations of embodied cognitive science. Consider how multicellular agents are themselves constituted out of highly competent, cognitive units (Baluška and Levin, 2016; Levin, 2019, 2020, 2021; Levin and Dennett, 2020; Lyon et al., 2021). In other words, the cognitive cogency of the higher-level (in this case, multicellular) agent depends on the scaling up (see Section How fine-grained functional details matter to cognition) of the cognitive processes—agency, goal-directedness, decision-making, memory, learning—found in the dynamics of constituent (somatic) cells. As we will see (in Section Soft robotics), individual cells are remarkable structures that, due to their regulatory and organizational dynamics, maintain internal milieu viability and their connectivity with other cells in the extracellular tissue complex with precision and flexibility. Reminiscent of 19^th^ century theories of the “cell state” (Reynolds, 2007), our approach thus positions organismic cognition as emblematic of the homeostatic and self-organizing processes that typify all living units. Or, as Man and Damasio put it, “high-level cognition [is] an outgrowth of resources that originated to solve the ancient biological problem of homeostasis” (Man and Damasio, 2019: 447)—hence, cognition all the way down.^3^

As it happens, the building of higher-level cognitive agents out of progressively smaller—but still cognitive—units and parts is precisely the perspective being taken up in the domain of soft robotics and synthetic biology. Ebrahimkhani and Levin (2021) provide a flavour of this style of argumentation:

“One feature of bioengineering at the meso-scale that is unique… is the fact that bioengineers build out of parts that are themselves highly competent, for example, cells that have their own internal homeostatic and signalling systems. Thus, the experiments that are done with biological parts have the potential to help understand how swarm intelligence plays out at the tissue level to solve morphogenetic problems. Such advances… act as an inspiration for novel architectures in machine learning, artificial intelligence, and resilient autonomous swarm robotics.”

Indeed, this bio-inspired approach feeds well into current ambitions of developing AR and unconventional computing platforms (e.g., Jones, 2015). The key is how the above sciences emphasise the importance of an *active matter* approach. Rather than the inert, hard, and passive parts traditionally used in robotics, active matter approaches indicate how the very materiality of the system can perform complex feats that obviate the need for overarching or centralised control (Bechtel and Bich, 2021; Kaspar et al., 2021). In what follows, we survey the fields of active matter physics and soft robotics to then return in Section How fine-grained functional details matter to cognition to our fine-grained functionalist take on how the matter matters for life and cognition. By now, it should be clear that in arguing this position we are not being substantialists: it is not this or that type of matter (say, carbon) that is important, but the matter insofar as it can sustain organizational complexity of the right sort.

### Active matter

Active matter physics (AMP) is a vibrant field of research that has received significant attention in recent decades (see Baez, 2021). Theoretically, it sits at the intersection of physics and biology and deals with materials and material systems that are intrinsically out of thermodynamic equilibrium. Some examples of these are field-responsive matter, hydrogels, and piezoelectric materials, while active matter systems are those that harness properties of such materials to drive their distinctive non-equilibrium behaviour. These include the actomyosin cytoskeleton (Needleman and Dogic, 2017; Jülicher et al., 2018), cellular activities (Fodor and Marchetti, 2018), swarming behaviour (biofilms, multicellular bodies: Wioland et al., 2016; Kempf et al., 2019), and even macroscale organizations such as avian murmurations and herds of animals (Cichos et al., 2020). Although this might appear to be a heterogeneous set, these systems exhibit the broad commonality that their individual units (motor proteins, cells, individual organisms) are themselves highly competent, active contributors to group dynamics (Needleman and Dogic, 2017).

For example, it is increasingly common to view multicellular bodies as a kind of swarm behaviour (Arias Del Angel et al., 2020), which depends on the intrinsically active nature of constituent cells. Indeed, Arias Del Angel et al. (2020) have commented on how facultative multicellularity in both protists and prokaryotes depends on active, field-responsive, and internally driven physical processes of constituent parts, remarking that the overall organismic form hinges on the interplay of the inherent physical properties and agent-like competency of cells making decisions in a context-sensitive and flexible manner. In contrast, then, to passive systems (e.g., the Rayleigh-Bénard cell) that receive energy exogenously at a boundary condition, active matter systems—of which organisms and certain designed systems are paragons—themselves consist of units that are internally driven (Batterman, 2021). Crucially, Needleman and Dogic (2017) remark that active units are capable of *self-organization*, whereas passive units can only *self-assemble*. In the context of being “suitably organized” what we see is that not any back-of-the-envelope set of materials can sustain the organization dynamics on which life and, we add, cognitive processes depend—instead, to be suitably organized one must have self-organization, and it is here that an active matter approach is most pertinent.

Seeing how the dichotomy of active and passive structures underpins much of the literature in AMP, it is worth explicating further what marks out the former exactly. In their influential review of AMP, Marchetti et al. (2013) write that active matter systems consist of the following features:

“They are composed of self-driven units… each capable of converting stored or ambient free energy into systematic movement. The interaction of the active particles with each other, and with the medium they live in, give rise to highly correlated collective motion and mechanical stress. Active particles are generally elongated and their direction of self-propulsion is set by their own anisotropy rather than fixed by an external field (Marchetti et al., 2013: 1144).”

The key distinction we wish to draw out here is that being an active matter system relies on two features: (i) the energetic nature of the constituent units (actively converting ambient energy as opposed to being driven solely by energetic contributions at an external boundary conditions) and (ii) their inherent shape (anisotropy) influencing the systematicity or directionality of how energy is used—“geometry of [its] interface shape can control sensitivity to the environment” (Hanczyc and Ikegami, 2010). The example of the Rayleigh-Bénard cell will help draw out the important difference between the two types of material configurations (i.e., passive vs. active).

Rayleigh-Benard cells are a paradigmatic case of non-equilibrium activity. These are familiar to anyone who has ever added cool oil or water to an evenly heated pan. The sudden encounter of the droplets with a hot surface drives a phase transition that constrains the activity of individual water molecules. The result is a highly ordered hexagonal structure that is continuously sustained so long as energy input is consistent. Some commentators remark on how structures like the Rayleigh-Bénard cell represent the precursor dynamics from which goal-directedness endogenously emerges (Juarrero, 2015), and they are hence common reference points in discussions of emergence, agency, and goal-directedness (cf. Moreno and Mossio, 2015).

Despite its relevance as a model for far-from-equilibrium processes, the Rayleigh-Bénard cell does not qualify as an example of an active matter system. The reason for this has already been suggested above, but Needleman and Dogic make it clear: “Rayleigh-Bénard patterns are non-equilibrium dissipative structures, but each convection roll is composed of passive molecules, and the entire system is driven away from equilibrium by energy provided through an external macroscopic boundary” (Needleman and Dogic, 2017: 1-2). They meet neither requirement (i), as they are composed of energetically passive molecules, nor requirement (ii), as their shape is a result of the motion guided by an external energy gradient, in no way dependent on inherent properties of the medium itself—which, without influence, would immediately relax into an amorphous shape as normally water molecules on a pan tend to do. Contrastively, active matter physics addresses the activity of thousands of nanoscale molecular motors that interact to create mesoscale, self-organizing structures. Common examples span the living and non-living domains, including model systems such as self-propelled oil droplets (Hanczyc and Ikegami, 2010; Hanczyc, 2011; Cejkova et al., 2014), active microtubule networks (Sanchez et al., 2012), cytoplasmic flow (Mogilner and Manhart, 2018), and the eukaryotic cytoskeleton (Brugues and Needleman, 2014). More recently, active materials have been exploited in soft robotics (Ebrahimkhani and Levin, 2021), computer science (Jones, 2015), and AI (Kaspar et al., 2021) as a way to overcome the many resource constraints that have long plagued the fields. The key point can be expressed as follows: “The cellular cytoskeleton, cells, and entire tissues [as exemplary active materials] are driven away from equilibrium by the continuous motion of thousands of constituent nanoscale molecular motors, protein-based machines that transform chemical energy into mechanical motion” (Needleman and Dogic, 2017: 2). Intriguingly, this is a point that has echoes in Section Traditional vs. fine-grained functionalism in our discussion of fine-grained functionalism and the relevance of spatial *scale*: “Metabolic processes in cells occur at a specific spatial scale, the scale measured in nanometres… In that context and at that scale, matter behaves differently than how it behaves elsewhere. … There is unending spontaneous motion that does not need to be powered by anything external” (Godfrey-Smith, 2016a: 485). At larger, more coarse-grained scales, these complex and systematic processes do not occur. Already, then, we come to see how fine-grained structural details matter for sustaining self-organizing dynamics at a wider variety of scales.

Of course, what is central to the discussion of fine-grained functionalism is the connection between these active material processes and prototypical instances of cognition, such as goal-directedness, memory, learning, agency, systematic directionality, and so on. As it happens, recent work on active materials has begun to show the variety of ways in which some individual—and sometimes multiple—capacities are present in non-living systems, a discovery that has led some to speculate that AMP is revealing not only the physics of life (Popkin, 2016), but the physics of cognition as well (McGivern, 2020). To wrap up the discussion of AMP, then, we make a more direct connection to work on basal cognition and the concept of existential needs introduced above.

Capacities of non-living active matter systems that have been particularly illuminating are those of autonomous movement, environmental sensing, coordinated action, and problem solving (McGivern, 2020). The ability to accomplish these feats importantly depends on the material situation of both the system in question (swarming nanobots, self-propelled oil droplets) and the environment where it finds itself. In self-propelled oil droplets, for example, researchers introduce internal convection currents that create a bifurcation between systematic internal activity and its viscous medium. The droplet's movement is driven by a convective flow that has an uneven influence on the inside of the droplet, which helps create a feedback system between its internal dynamics and the medium external to it (so-called Marangoni flows; see Hanczyc and Ikegami, 2010 for a review). Although this is a simple system, Hanczyc and Ikegami (2010) suggest that it serves as a model system for understanding the origins of chemotaxis in unicellular organisms, as the droplet must continuously navigate gradients to find the chemicals that sustain its internally driven dynamics: “The system becomes sustainable by circulating the reactants and products effectively as organized by the convective flow” (Hanczyc and Ikegami, 2010: 236). As we can see, it meets both conditions of active matter listed above: (i) its constituent particles are internally energetically driven in that they tap into reservoirs of (Gibbs) free energy available within the system, and (ii) its droplet shape results from the inherent properties of the oil (its viscosity and surface tension) and its relation to the medium in which it is embedded; moreover, the geometric configuration of the oil droplet actively contributes to the distinctive capacities it exhibits. This is an intriguing model system for our purposes, as it places a premium on active materials and constituent units that spontaneously self-organize and, given the right guidance and influence from designed experimental parameters, can sustain itself for significant periods of time. Although not elaborated here, the case of oil-droplets also underlines the way in which the inherent shape (“geometry-induced fluctuations”; Hanczyc and Ikegami, 2010) of an active unit determines locomotion and, in bacteria, chemotaxis. Moreover, a mechanical pushing of the cytoplasmic sol of a cell (as in the social amoeba *Dictyostelium*) elicits directional and coordinated motion (see Dalous et al., 2008; Boussard et al., 2021). Thus, the properties exhibited in active matter systems, such as oil droplets, highlight the material basis for capacities found throughout the living domain.

AMP shifts our focus on the study and development of minimally cognitive systems (that is, systems that exhibit prototypical features of cognition such as directional locomotion, memory, or learning) in two important ways. First, it does not aim to replicate paradigmatically intelligent behaviour modelled on human activity (playing chess, say) and instead emphasises environmentally embedded behaviour with wide distribution in the natural world (McGivern, 2020). Secondly, and perhaps most centrally for our argument on MR 2.0, “work on active materials is not specifically aimed at computational characterisations of behaviour” (McGivern, 2020: 442), i.e., it does not rely on coarse-grained functions of the medium of interest, but rather builds on simpler, well-established principles from areas such as condensed matter physics, building a bottom-up description of activities of systems of interest. Work within AMP, then, demonstrates how harnessing the physical processes and active materials that underlie organismic behaviour contributes to and mediates the cognitive sophistication we find in the biological domain—suggestive of how such principles can, and are, being exploited in the domain of artificial and designed soft robotic systems, which we turn to shortly.

Before continuing, however, it is worth dwelling on the aspect of AMP that we see as central to the discussion of cognition that forms the remainder of this paper. Recall from the introduction that our understanding of cognition revolves around the fulcrum of existential needs and how capacities such as agency, goal-directedness, and self-maintenance are the basis for further cognitive sophistication. Matthew Egbert has recently argued that non-biological model systems—such as our humble oil droplet—serve as ideal testbeds for exploring the material and thermodynamic basis of these existential needs: “conditions that must be met for [that system] to persist and… behave in ways that satisfy those needs” (Egbert, 2021: 5).

There are two ways to understand existential needs vis-à-vis any object or system, the first rather banal and the second more critical for the kinds of systems we explore in this paper. The first is the sense in which, trivially, any object must have existential needs to be what it is. A table cannot be heated above a certain temperature or subject to a certain amount of pressure and still remain a table. But, and this is the more important point, there are crucial differences between what is required of garden-variety non-dissipative objects like rocks, tables, and chairs to be what they are and self-organizing, self-maintaining dissipative systems in far-from-equilibrium conditions. The difference in existential needs for the two types of systems is captured as follows:

“[Non-dissipative entities] are merely passively stable, whereas dissipative structures are constantly falling apart and yet persist thanks to processes of repair, replacement, or reconstruction. This means that existence for passively stable entities is the absence of a destructive event. In contrast, for dissipative structures, existing is a process—and a process that must continue for the system to persist (Egbert, 2021: 5).”

Importantly, processes have quantifiable and measurable rates that open dissipative structures to a study of how viable such a system is, that is, how well it persists despite the tendency to degrade. As Egbert notes, there is no equivalent measurement for passively stable systems: their existence is not a process and does not require the same set of behaviours and activities that active matter systems engage in. We can therefore agree with Godfrey-Smith when he writes “macroscopic machines provide a poor model for the material basis of living activity and for the material basis of mental activity in living beings like us” (Godfrey-Smith, 2016a: 489).

Our discussion of AMP furnishes one strand of the argument for fine-grained functionalism, namely, that the fine-grained material and thermodynamic details of living systems matter a great deal more than common assumptions on the MR of the cognitive might *prima facie* suggest. Indeed, organisms are subject to what physicists call the “tyranny of scales”—they are sensitive to, and influenced at, every order of scale, from the nanoscale to the mesoscale, and for multicellular agents like ourselves, the macroscopic scale. These are highly sensitive coordinated structures, and there is no non-arbitrary point below which the physics no longer matters to manifesting the distinctive cognitive capacities that contribute to a living system's survival. Although our discussion of cognition has been minimal in this section, we turn now to soft robotics to see how these insights are being actively taken up in designing intelligent synthetic machines.

### Soft robotics

Soft robotics is a sub-discipline of robotics and artificial intelligence that explores how intelligent, adaptive, and plastic behaviour emerges out of the inherently active, precarious, and soft parts that constitute such systems. It is a discipline that examines how to construct systems that exploit the physical laws and tendencies at play at every level of scale. In other words, it investigates how organisms are embedded and subjected to a “tyranny of scales” that must be accommodated and exploited to meet their existential needs (Ebrahimkhani and Levin, 2021) and how one may apply these insights to the creation of intelligent machines.

Tellingly, Shah et al. remark that the inspiration for soft bodied robots comes from the highly integrated nature of biological cognition: “In these integrated living systems, intelligence, memory, learning, behaviour, and body structure are all intertwined and emerge from the multiscale dynamics of the same robust and highly fault-tolerant medium” (Shah et al., 2021: 1). This is put in contrast with the standard hard (and passive) components that constitute more standard roboticist approaches. Standard approaches have had some success in the form of modular parts that can be added or taken away depending on the task (such as passive conforming grippers and certain algorithms that can re-adapt to distinct tasks). But even in cases where these techniques might achieve certain adaptive ends, they “operate under the assumption that the robot's body is only reconfigured or reshaped due to external forces, and do not explore the possibility of synthetic machines that actively grow, regenerate, deform, or otherwise change the resting shape of their constituent components” (Shah et al., 2021: 2). Contrastively, as we saw above, the field of AMP begins to highlight the way in which organism morphologies and bodies are inherently active structures that respond proactively to changing environmental situations—a form of adaptiveness that depends crucially on the highly active processes that comprise cellular structures, multicellular integration, and cognitive capacities such as goal-directedness and agency. This picks up on a point made earlier: a key feature of organismic cognition and an insight that has been actively taken up in soft robotics is that higher-level cognition relies on constituent parts that are themselves *highly* competent.

Here, we focus on how the concept of existential needs, raised in the introduction, is critical for the creation of artificial machines capable of autonomously selecting actions required for self-maintenance. In other words, in contrast to passively maintained robots that must be externally guided and directed toward goals, tasks, or functions, it is suggested that (i) the inherent vulnerability of soft embodiment coupled with (ii) thermodynamic processes that are required to maintain a system in such a state would endow an artificial agent with the kind of autonomous self-maintenance and self-organization that are important for cognition (cf. Bickhard, 1993). Only then would these designed systems have real “skin in the game” (cf. Bongard and Levin, 2021). To put the matter differently, to design machines capable of autonomous decision-making, behaviours must have *consequences* for how the system can and should act in the world. We have introduced this idea previously in terms of precarity and risk-to-self (in Section Introduction), and with the analysis of active matter above we may further specify the details of what precarity would mean for a machine. Man and Damasio (2019) indicate, in terms intriguingly close to Egbert's paper cited above, that we can understand how feelings emerge from a physiological investigation of life regulation. Feelings, they argue, are not sufficiently approximated by arbitrary reward or loss functions of standard approaches to AI, since the worldly risks and consequences should directly impact the continued existence of the machine. The quality of feeling “is the harbinger of the good or bad outcome relative to survival” (Man and Damasio, 2019: 446). They argue—and we concur—that it is only at the point when the machine can consistently strive for continued existence that true agency may arise.

It is important to note that what we mean here by “life regulation” is not biology-restricted, but rather is the upshot of a far-from-equilibrium system working against the tendency toward dispersal. The suggestion we would like to make here is that to be this kind of system—a system for which there can be situations that matter to it—it must be a self-organizing one constituted by active physical processes inherent to the materiality of the system in question. Soft robotics, then, is in the business of identifying how the material aspects of the body exploit physical laws to expand robot functionality (Shah et al., 2021: 2).

For example, Pishvar and Harne (2020) note that soft robotics incorporates field-responsive smart matter that can induce an internal flux in response to an applied field that tailors material characteristics of the media, influencing its function and behaviour. As they write, “When responding to applied fields, a multitude of internal changes are possible in soft, smart matter” (Pishvar and Harne, 2020: 1). The range of adaptability is thus expanded when one incorporates material properties that are themselves active contributors to overall robot functionality, in contrast to standard hard parts used in robotics, whose adaptability—in the rare cases when they are adaptable—is due to pressure driven forces at an external boundary condition. More recently, Kaspar et al. have argued that “synthetic matter that itself shows basic features of intelligence would constitute an entirely new concept for AI” (Kaspar et al., 2021: 345). They dub this pivot in AI and robotics the “rise of intelligent matter” and reiterate the point that incorporating active materials into AI and robotics programmes would expand robot functionality “far beyond the properties of static matter” (Kaspar et al., 2021: 345). Examples of such smart, active matter systems include artificial thermoregulating skin (Kanao et al., 2015), emergent swarming activity of concerted nanobots (Wu et al., 2021), and xenobots that sit at the intersection of bio- and artificial engineering (Ebrahimkhani and Levin, 2021; Kriegman et al., 2020, 2021; Blackiston et al., 2021). All authors appear to be in agreement that incorporating the smart and active propensities of soft matter is crucial for achieving autonomous behaviour in the domain of robotics (Pishvar and Harne, 2020; Kaspar et al., 2021)—a suggestion we will turn toward in the next section on the fine-grained functionalism approach to AR.

### Summary

To wrap up briefly, the fields of active matter physics and soft robotics are working lock and step to uncover the diverse functionality, adaptability, and plasticity inherent to certain materials that remain in far-from-equilibrium conditions. They are thus fields that explicitly consider the thermodynamic situation of the system in question. An upshot of this is that not any sort of material can accomplish the diverse behaviour or cognitive sophistication exhibited in the biological domain. In other words, to be “suitably organized” requires attention to the media out of which the system of interest is constituted: the matter matters for cognition and is not a dimension of robot functionality that can be abstracted out. Indeed, the conclusion we wish to draw from this literature is that *more* attention should be paid to the material basis of cognition than is commonly done.

Given the importance of the above two testbeds for exploring the nature of cognition, it is crucial to explicitly articulate the connections that can be drawn between active matter physics and soft robotics. Our reasoning for progressing from the former to the latter is that active matter physics deals with far-from-equilibrium dynamical systems, writ large, and the materials and material constellations that can sustain self-organizing processes on time scales relevant to the biological world. It is precisely these processes that are then exploited and harnessed in guided assembly to arrive at the sophistication we find in the field of soft robotics (Ebrahimkhani and Levin, 2021). While prima facie it might appear that the two fields can work in isolation, Pfeifer et al. suggest why this is not advisable: “it [is] clear that autonomous agents display self-organization and emergence at multiple levels: at the level of induction of sensory stimulation, movement generation, exploitation of morphological and material properties, and interaction between individual modules and entire agents” (Pfeifer et al., 2007: 1088)^4^. In other words, active matter physics in tandem with soft robotics furnishes not only the empirical testbeds for crafting more sophisticated autonomous agents, but also renders tractable notions of emergence and self-organization that are of relevance not only to bodily maintenance and self-preservation, but also cognition and the behaviour required to keep such systems viable. In the following section, then, we dovetail the pieces of the argument laid out thus far to advocate for an emerging approach to the development of AI and AR that stems from fine-grained functionalism suggested in Section Traditional vs. fine-grained functionalism and the results of AMP and soft robotics discussed in the current section; a paradigm that appreciates the importance of the materiality of cognition.

## How fine-grained functional details matter to cognition

In this paper, we explore the possibility of developing autonomous robots capable of prototypical forms of valuing and engaging with the world in a goal-directed manner. To this end, we adopted the notion of precarity (i.e., “risk-to-self”) and focused on the existential needs of a system to remain in a far-from-equilibrium state. We saw that a step in this direction requires rethinking some of our basic assumptions regarding matter and its relation to cognition, which remain deeply embedded in existing approaches to the mind and brain sciences, as well as in approaches to AI and robotics. Indeed, rather than a “layered-cake” model of levels that renders higher-level cognitive phenomena as resting autonomously from and “on top” of its substrate (i.e., “hardware”), we set out to complicate this picture by emphasising the (bio)physical nature of the structures that support, enable, and implement cognition. What we want to suggest is that more attention must be paid to the *fine-grained* details of the system when understanding and studying cognition—and then recreating it in alternative media. This is the crux of the fine-grained functionalism introduced in Section Traditional vs. fine-grained functionalism. Although it is common to see biologists and philosophers emphasise the “Heraclitean” nature of biological matter and metabolism, we believe recourse to the fields of active matter physics and soft robotics situates fine-grained functionalist views on a sturdied empirical testbed. Thus, the developments in these disciplines enable an exploration and substantiation of claims about precarity and existential needs vis-à-vis cognition, which so far we have explored mostly in the abstract.

In this section, we weave the threads of the argument together to argue that creating AR capable of valence and goal-directedness requires us to think about the organizational and material dynamics of the embodied system in a more thoroughgoing way. In other words, what we are suggesting is not (or not simply) that the body *simpliciter* matters to cognition, as advocates of sensorimotor coordination have long held (see Van Duijn et al., 2006). Rather, we argue for a multiscale account in which cognitive and agent-like competency is present at nearly every level of a biological heterarchy capable of sustaining the appropriate organization—cells, tissues, networks, the whole organism, and even swarming behaviour of eusocial species (Levin, 2019). In contrast to views that situate cognition as exclusively proprietary to a higher-level organism, we present an account in which the scale and “selfhood” of the cognitive agent are highly malleable, plastic, and vacillatory.

We therefore argue for taking embodied approaches further than is commonly done in two important respects. First, in the multiscale approach just outlined: higher-level systems (organisms or future soft robots) themselves consist of highly active and competent cognitive units. The preservation of cognitive functionality at varying spatiotemporal scales is indeed a crucial aspect of evolvability and robustness in organisms (Levin, 2020). Cognition is then regarded as “an outgrowth of resources that originated to solve the ancient biological problem of homeostasis” (Man and Damasio, 2019: 447). It is construed as an activity of self-organizing and self-maintaining processes fundamental to all living organisms, and that is then appropriately scaled up throughout a biological heterarchy, an idea we explore further below. Second, and here we loop back to fine-grained functionalism, these cognitive capacities depend crucially on the material and (bio)physical details that are standardly abstracted out or relegated to a “hardware” problem. These two central themes are discussed in the remainder of this section.

### Scaling cognition all the way down—and back up again

Organismic embodiment is characterised by highly plastic and adaptive parts responding in a coordinated manner to wider organism-level goals. Empirically, this increasingly seems to rely on essentially cognitive units and intelligent parts—i.e., cells, tissues, networks—acting in a concerted manner that involves an “inter-penetrating, concurrent operation of numerous layers of cognition within the same living system” (Levin, 2021: 4); that is, it involves cognitive units maintaining some degree of flexibility, agency, and goal-directedness that is executed in local and global contexts. In biology, this is often on display in the morphogenetic and ontogenetic unfolding of the organism—a complex process that requires cognition to be tailored to both scale-specific as well as scale-free needs in regulating organism development. Indeed, the ability of organisms to plastically change shape throughout their life cycle is the current envy of soft roboticists, where developing shape-changing robots is a frontier in the field (Shah et al., 2021). Here, we explore the phenomenon of shape-shifting, as it helps illustrate how morphogenetic and homeostatic goals pursued at each level can give rise to robust and flexible behaviour at a variety of scales.

As suggested, an organism's ability to arrive at complex morphogenetic outcomes depends on the interpenetration of these functionalities at a range of spatial and temporal scales, as well as the elasticity and robustness of a (predominantly soft) medium. This contrasts with standard roboticist approaches that incorporate hard parts [“up-armouring”, as Man and Damasio (2019) phrase it] and assumes that bodies are only reconfigured due to external forces, effectively neglecting the active and proactive responsiveness that typifies biological media and serves as the foundation for homeostatic, self-organizing processes. Indeed, hard-clad robots might experience change at the movement of a joint, but none within the stuff that constitutes it. Contrastively, biological and soft robotics systems “[change] shape at all relevant scales, globally and locally” (Shah et al., 2021: 10). What is important here is that this process is effectuated through the nested hierarchical structure in which every level can pursue its own local (morphogenetic) goals. The morphogenetic (shape-shifting) outcome of this process is thus not only materially and physically active, but an expression of the cognitive coordination to be found throughout the organism. This is in sharp contrast with current robotics, which largely uses unintelligent parts (Shah et al., 2021).

We can call these systems exemplars of “coordinated structures”, following Kelso (2016), which are endogenously self-organized systems determined by their own dynamics. Indeed, a characteristic feature of such structures is that they do not depend on an exogenous “ordering influence” (Kelso, 2016: 491), and some have remarked on how this form of self-organization is the basis for higher-level features of autonomy, agency, and goal-directedness (Juarrero, 2015). Perhaps unsurprisingly, the requirements for coordinated structures are parallel to the defining features of active matter systems, suggestive of the relevant building blocks for engineering artificial analogs that could come to endogenously self-organize to create novel, agentic, and goal-directed structures. It should be clear that the vision of cognition we have in mind here is one in which the system itself has real “skin in the game”, and therefore requires this minimum degree of autonomy (Bechtel and Bich, 2021).

Importantly, this focus on internal coordination echoes the prominent view that cognition—as it evolved—initially emerged in the course of evolution for coordinating cellular metabolisms and ultimately multicellular (more minimally, intercellular) activity, particularly spatial and temporal coordination across parts of the system—a position Keijzer et al. call the “Skin Brain thesis” (Keijzer et al., 2013; Jékely et al., 2015). The primacy of internal coordination hints at the profound relevance of electrical oscillatory activity found in biological bodies (cf. SELFOs, see Hanson, 2021), which has been put forward as one of the central mechanisms of synchronization—an important topic that future work will explore in detail. Furthermore, the path of engineering intelligent systems from self-organizing and coordinating intelligent parts, while perhaps not the only one, becomes a clearly feasible approach for researchers, since we see that in the history of life this trajectory has in fact led to the emergence of cognition.

The shifting local and global coordination of the system (i.e., organism) exemplifies the distributed approach to cognition we have defended herein. It requires that constituent cells and parts maintain certain aspects of cognitive function—memory, learning, agency, decision-making—at least in the service of their own form and function. Levin captures this point well when he writes that “somatic cells did not *lose* their behavioural plasticity … to become parts of metazoan swarms (bodies): they scaled them to enable pursuit of larger goals consisting of creation and upkeep of massively complex anatomies” (Levin, 2019: 5).

Thus, the concept of scaling up, which we have relied on throughout this paper, rests on the idea that multicellularity is itself a highly complex and competitive “environment” that requires local and global morphogenetic goals consisting of trade-offs and top-down constraints between small-scale outcomes and organismic level development. We have already suggested that we can understand this principle of scaling up in terms of internal coordination determined by endogenous dynamics but building on the concept of active matter can help elaborate the idea further.

We can identify an appropriately scaled-up active matter system when two conditions are met: (1) the system is not wholly determined by local causes (see Kelso, 2016), that is, the system behaves in relation to non-local causes; and (2) it exhibits goal-directedness as a coherent unit. Internal coordination results from the conjunction of these conditions and, hence, is inseparable from cognition as we explore it here. Our reliance on active matter is motivated by our fine-grained functionalist claims, which we turn to below.

For now, it is important to emphasise that developmental bioelectricity has been identified as the predominant mechanism behind locally and globally coordinated morphogenetic, developmental, and cognitive outcomes—realising the scaling up of parts into wholes. Bioelectrically coordinated and integrated cells (in the form of an organism or a colony of organisms, as in bacterial biofilms) meet the conditions for a scaled up system laid out above in that (1) the activity of cells often results from information about occurrences happening in a distant part of the integrated system and (2) each part coordinates its actions with others, so that the system as a whole exhibits a consistent behavioural pattern (see Arias Del Angel et al., 2020 for an insightful discussion of this in relation to the social amoeba *Dictyostelium*). Thus, the bioelectrical activity that has often been associated with nervous activity is increasingly seen as an exploitation of highly preserved, ancient, and widely distributed cellular functions and capacities (Prindle et al., 2015)—and we extend the discussion to suggest that this itself hinges on more general properties of cellular, biological, and living material dynamics. This is what in developmental biology (Newman, 2019, 2022, 2021) has been called “biogeneric” processes, indicative of how biological functionality in the service of homeostatic, morphogenetic, and developmental goals is an exploitation of general physical principles of viscoelastic media and oscillatory activity. This again draws a strong connection between the “physics of life” and “physics of cognition” we hinted at above.

Indeed, if—as this research suggests—neurons are specialised exploitations of bioelectrical mechanisms, it is more fitting to see the nervous system as initially (both *evolutionarily* and *ontogenetically*) more a matter of “pulling the organism together” than as specialised for higher-level cognitive functions (cf. Fields et al., 2020). Again, pulling from Levin, we see that “neural networks control the movement of a body in three-dimensional space; this scheme may be an evolutionary exaptation and speed-optimization of a more ancient, slower role of bioelectrical signalling: the movement of body configuration through anatomical morphospace during embryogenesis, repair, and remodelling” (Levin, 2019: 5).

The truly innovative move in the literature on basal cognition (that is, cognition as situated in more “primitive” organisms and cellular activities), then, is the explicit recognition of the cognitive (or proto-cognitive; Godfrey-Smith, 2016a,b, 2017) nature of the activities identified above. Indeed, examples of memory in social bacteria (Dinet et al., 2021), learning in unicellulars and protists (Gershman et al., 2021), decision-making in acellular and cellular slime moulds (Arias Del Angel et al., 2020; Smith-Ferguson and Beekman, 2020; Boussard et al., 2021) have all been identified in non-neural organisms, and it is known that constituent cells in metazoan swarms (i.e., multicellular animals) actively and adaptively manage their morphology, behaviour, and physiology as needed for survival. Again, this is cognition within and throughout biological bodies and therefore is suggestive of a more thoroughly embodied cognition insofar as higher-order organized wholes are dependent on constituent units and parts maintaining, in certain crucial respects, cognitive capacities of far more ancient origins. The ability of evolution and hence organisms to exploit the material properties of cellular processes to yield coordinated wholes is the current envy of soft robotics approaches that still rely on guided self-assembly to arrive at robot functionality (Ebrahimkhani and Levin, 2021).

As we have already hinted in Section Traditional vs. fine-grained functionalism, this differs dramatically from extant AI and robotics approaches that do not avail themselves of such techniques, in that goals can be pursued both at the wider level of the whole organism, at the tissue complex level, and the level of cellular homeostasis and intercellular coordination. Indeed, “the ability of each nested level to have its own local morphogenetic goals… contrasts with today's robots, which are largely made of unintelligent parts” (Shah et al., 2021: 10). To conclude, then, we loop back to our fine-grained functionalism claims to highlight the close imbrication between cognitive capacities of interest in the design of AR (agency, goal-directedness, memory, learning, self-maintenance) and fine-grained aspects of the materials that should, we suggest, be the focus of current and coming robotics approaches.

### Fine-grained functions of soft materials

Fine-grained functionalism rests on the crucial observation that cognition is not temperature. Allow us to explain. When we approach cognitive systems and try to individuate the functions they perform, we always do so at a particular level of granularity that reflects certain aspects of the observer (their interests, needs, pragmatics, assumptions) and not the cognitive system observed. Philosopher of science Angela Potochnik phrases this as a matter of reading our assumptions of the multilevel nature of the world *into* the phenomena of investigation, imposing an artificial hierarchy on a complex system where there may not be one (cf. Bechtel and Bich, 2021; Potochnik, 2021). Crucially, these different granularities do not simply map onto the distinction between macro- and microstates that some branches of physics find useful. Cognitive functions, such as learning, memory, decision-making, are not macrostates realised by (possibly very different) physical microstates in the way that the same temperature can be realised by various distributions of thermal energy across molecules—even if we may observe these functions in equal part in a variety of natural and engineered systems.

What we mean to say, then, is that non-biological passive materials (in our case, materials that cannot sustain self-organizing dynamics in far-from-equilibrium conditions) will *not do* the same things as soft biological counterparts: “They will be functionally different, not merely different in “hardware” or “make up”' (Godfrey-Smith, 2016a: 501). For functional equivalence, their material structure and organization must occupy specific spatial and temporal scales to endogenously accomplish self-maintenance and self-organization. The main upshot of this view is that fine-grained functional properties of living systems, such as metabolism and recursive self-maintenance, matter quite a deal more than is commonly supposed in debates on the MR of the cognitive. In other words, if cognition depends on suitably organized, endogenously driven internal dynamics of cellular activity and the appropriate scaling up into even smarter wholes, we begin to see the way such details become the foundation of the cognitive—and, by extension, central to the approach to engineering AR that we advocate. There are two considerations with which we would like to conclude.

First, fine-grained functionalism imposes clear constraints on what sorts of materials are capable of instantiating cognition—without falling into the biological chauvinism typically (and erroneously) associated with this type of view. The required platform must be able to sustain self-organizing dynamics in far-from-equilibrium conditions on temporal and spatial scales that make it susceptible to physical forces, constraints, and tendencies that are not found at larger spatial scales—the scale of standard machines to which biological cognition is traditionally compared (Nicholson, 2014). These conditions are met by soft, active materials: a domain of materials science that continues to grow in popularity since its inception in the 1990s. From a physicist's perspective, exemplary soft materials such as “[c]olloids, polymers and surfactants, sometimes also known as ‘complex fluids’, have one characteristic in common: they involve a mesoscopic length scale between the atomic (~1 nm) and the bulk (~1 mm). On this intermediate length scale, one finds structures such as suspended particles/droplets, macromolecular coils, and self-assembled structures such as micelles and bilayers” (Poon, 2000). The ability to self-assemble into vesicles is especially interesting, as, according to some researchers (see Kauffman, 1993), such structures form a necessary step in the emergence of life, since they allow for the prebiotic system enclosed within to control its interactions with molecules in the environment and, in result, to remain at the boundary between subcritical and supracritical behaviour. As stated throughout, we do not preclude the possibility of non-living cognitive systems. Indeed, crucial points of our argument turn on the blurring of cherished distinctions between paradigmatically living and non-living systems.

What we do want to highlight, however, is that a non-living system is cognitive the more it approximates dynamical features of living activity—that such activities (which we broadly associate with self-maintenance and, eventually, homeostasis *via* metabolic activity) are the fount from which higher-level cognition emerges. The overlap in the dynamics between living and non-living systems constrains the types of materials that can enter a concerted organization able to sustain itself recursively and endogenously. While common reference points in philosophical debates on MR 1.0 consist of cognition being instantiated by (*inter alia*) tumbling beer bottles, frenzied radio signalling between denizens of the Chinese nation, and, of course, Swiss cheese, it is clear from what we have argued that these are *not* the kinds of things that can sustain self-organization endogenously. Matter behaves differently at the scale of objects normally invoked to support intuitions on MR, reaffirming the point expressed above that these materials will not do the same thing as the molecular motors, nanoscale molecules, and field-responsive materials we find at length scales well below that of everyday familiarity. Dislodging our intuitions about the MR of the cognitive, and upgrading from MR 1.0 to MR 2.0, enables us to attentively observe the behaviour of matter at nano and mesoscales to more properly assess how proto-cognitive capacities relate to the frenzied activity of fine-grained features of the system—not treating them as “noise” or obfuscating complexity to be abstracted out.

What we do want to highlight, then, is that an active matter approach oriented around soft materials could begin to approximate these features in non-living systems and media—as the exciting field of soft robotics is beginning to show (Section Soft robotics above). Hence, an attempt to design and engineer an artificial cognitive system in such materials would tend to fall closer to cognition than extant hard-part robotic systems. Soft materials and our active matter lens provide (some of) the resources to better assess the “suitably organized” claim so often made in debates on multiple realisability. As already mentioned, this allows us to resist biological chauvinism worries, while also delimiting the kinds and configurations of systems that can be autonomous cognitive agents, hence neutralising the liberalism charge as well. That is, as stated earlier, the active matter lens allows us to argue *both* that the materiality of cognition matters *and* that the cognitive can be realised in alternative media (Chirimuuta, 2018; Brunet and Halina, 2020).

These considerations bring us to the second important insight granted by the perspective of fine-grained functionalism. It constrains how we should approach the task of engineering AR, defining a feasible—at least so we hope—research strategy. Developing artificial cognition once we have rejected the “hardware/software” distinction renders the concept of Artificial General Intelligence (AGI)—a Holy Grail of present-day AI researchers (explicitly embraced by companies such as OpenAI and DeepMind)—misguided. AGI can be understood as “loosely speaking, AI systems that possess a reasonable degree of self-understanding and autonomous self-control, and have the ability to solve a variety of complex problems in a variety of contexts, and to learn to solve new problems that they didn't know about at the time of their creation” (Goertzel and Pennachin, 2007). The view that emerges from the “cognition all the way down” approach is that cognition is not “General” in this sense—cognition is not a single programme that can be applied to a variety of contexts, in the way that the programme MuZero (Schrittwieser et al., 2020) is able to master a variety of video and traditional games without explicit presentation of the rules. Rather, cognition results from the orchestration of a vast amount of single-purpose, specialised processes that co-depend on each other across spatial and temporal scales—single cells coming together into larger and larger ensembles. These processes undergo constant rearrangements and shifts, balancing on the boundary of criticality, striving to remain far from thermodynamic equilibrium. What “Generality” the system and its parts exhibit results from constant flux, from its Heraclitean nature, where constant change is required to remain in the same place.

Hence, the task of engineering AR must not be approached from the top-down, and not only because of the high computational complexity of coarse-grained cognitive functions (discussed in Section Traditional vs. fine-grained functionalism). Soft materials need to be engineered into simple “proto-cognitive” units which then need to be scaled up into higher-level systems. While we believe that to a large degree appropriate scaling up requires self-organization, the researcher still remains largely in control of this process, as they can influence and shape the fitness landscape of emerging autonomous embodied robots, guiding them toward meta-stable states that they deem beneficial or useful. In fact, to a degree even an external re-arrangement of the emerging self-organized system may be enough to push it in a particular direction, in a manner similar to how surgical intervention into grown tissue makes possible the creation of xenobots (Kriegman et al., 2020).

This would mean that the task of developing AR doing a wide range of things—whether that would be driving cars, repairing spaceships, performing surgeries, or accompanying us at the table—is likely beyond the limits of what is attainable in the lifetime of the current generation of AI researchers. We believe, however, that the strategy remains similar whether one focuses on this kind of blue-sky research, or rather seeks to achieve more proximal goals that are already stated in the literature among the things engineers are working toward. These more feasible applications, specifically in the case of xenobots, include “intelligent drug delivery”, “internal surgery”, identifying cancer or processing of toxic waste products (listed by Kriegman et al., 2020), as well as “cleaning microfluidic chambers” and “environmental sensing” (suggested by Blackiston et al., 2021). The common approach to the development of such machines focuses on what conditions would be required for the system to believe this task to be “good”—not in terms of arbitrary reward functions, but in terms of risks and opportunities, or fitness landscapes. One way to accomplish this goal may be in parallel to raising and educating a child (cf. Ciaunica et al., 2021). In contrast to standard approaches in contemporary AI research, which may be more accurately compared with operant conditioning, raising a child consists more in creating—and removing—affordances in the social and physical environment of the baby. We create opportunities, control some of the risks, but in the end it is the child that must take up any particular affordance in order to best learn it. We reward, correct, and punish, but most often we do so implicitly, by accident, and to a much lesser degree than in the case of AI systems. These sparse rewards can be taken to serve more to structure the fitness landscape that the child explores, to boost its internal reward and motivation systems, than to provide a reward or loss function that learning can entirely depend upon.

The approach toward AR we suggest is similar. In the—paradoxically—simplest case where we rely on living soft materials as building blocks, we can observe an application of this strategy in the case of the aforementioned xenobots. In a virtual cyborg-like setup, they explore in simulation their expected fitness landscape guided by some simple tasks and then, *in vivo*, the simple self-organized structure is finessed through external means. The resulting living robot is capable of surprisingly complex behavioural feats, as it forages throughout its simple environment on a Petri dish, coordinating its behaviour with others, and—when presented with an opportunity of interacting with “naive” stem cells—replicating into active organisms, similar in form and abilities (Kriegman et al., 2021). Xenobots, in fact, offer an initial hint that an approach along the lines we suggested here is feasible and may well lead to the development of workable AR, even if with only limited applications to begin with.

### Summary

In sum, transitioning toward AR capable of selecting their own goals requires incorporating “dynamic materials that possess a substantial degree of conformational freedom, mobility, and exchange of nanoscale components” (Kaspar et al., 2021: 353). In other words, developing these autonomous systems calls for attending to the fine-grained functional profile of embodied active materials that do not themselves depend on exogenous control. This is in fact one of the main outstanding problems in synthetic biology and soft robotics, as current model systems are not capable of self-organizing in a coordinated manner across the nano and mesoscales, instead relying on researchers painstakingly guiding the process to a desired state. In point of fact, how organisms themselves are able to develop toward species-invariant morphological outcomes is itself an open question of momentous importance in biology. However, there is hope that exploiting what we already know about multicellular development and the physical principles of self-organization—paying attention to appropriate “scaling up” of intelligent parts into wholes—can help make way on this in the synthetic domain.

Our suggestion, then, follows recent lines of research that emphasise the importance of constructing machines that themselves comprise smart, active, and, in some cases, cognitive parts. Sometimes this is phrased as a matter of “off-loading” computation from centralised computers to the body, though the language of embodied computation is ambiguous and difficult to specify technically [see Nakajima et al. (2015) and Müller and Hoffman (2017) for divergent stances on this]. What does seem crucial for this next stage of designed soft robots is the ability to achieve global coordination in a more autonomous and self-organized manner, something currently out of reach but hopefully not for too long, as that is itself an active area of research.

## Conclusion

We opened this paper with the suggestion that to create autonomous embodied robots capable of valuing and engaging with the world in a goal-directed manner requires incorporating several dimensions of biological endowment. In particular, we looked at the notion of precarity (“risk-to-self”) and the related notion of existential needs. The proposal here has been that AI and AR come to autonomously value and interact with the world the more they approach biological analogs thereof–and the closer they approximate the dynamics that introduce the possibility of existential consequences for its actions. In other words, the proposal here has been to develop cognitive sophistication within alternative media by incorporating dimensions of vulnerability, precarity, and existential needs that emanate from the system's own internal dynamics with a denumerable set of actions that must be taken for this system to remain in far-from-equilibrium conditions.

To this end, we set out to dislodge several key assumptions embedded in the cognitive sciences that undermine the crucial role materiality plays in instantiating, mediating, and enabling cognitive form and function–specifically by proposing a fine-grained functionalist approach that treats the more minute properties of the system as central for cognitive function. The fields of active matter physics and soft robotics have begun to blur long-held dichotomies between hardware and software, living and non-living, machine and organism, and so on. But rather than reducing organisms to an anachronistic understanding of mechanism or matter, these fields have begun to actively question our understanding of materiality entirely. What we find, then, is not the hard, inert, and wholly passive parts standardly associated with machines and robots—but an inherent activity suffused throughout certain materials that, when brought into concerted, guided, and orchestrated engagement with one another *via* bioelectricity, can manifest and expand machine functionality in a manner unavailable to paradigms that do not avail themselves of these techniques. To construct autonomous robots, then, we propose an explicitly thermodynamic conception of life and mind that expands the domain of both terms. In effect, the view we have tried to articulate is one in which the mind is more material, and the material more mental, than is commonly believed—a view that is more at home in 18th and 19th century romanticist thought and American pragmatism (e.g., Charles Sanders Peirce) than it is with 20th century reductive theories of matter. When we shift our perspective away from one in which higher-level cognition sits across a divide from inert, passive matter to a view in which materiality is already pregnant with the possibility of the mental, we believe we move one step closer to the goal of creating autonomous and hence actually intelligent machines.

## Data availability statement

The original contributions presented in the study are included in the article/supplementary material, further inquiries can be directed to the corresponding author/s.

## Author contributions

DH, WR, and UL have equally conceived and developed the main thesis of the paper and edited, prepared, and accepted the final draft for publication. DH and WR wrote the first draft. All authors contributed to the article and approved the submitted version.

## Footnotes

^1^We are grateful to an anonymous Reviewer for pointing out that the ontological and epistemological interpretations of the claims of functionalism and MR are often conflated and prompting us to clarify our position. One could take this argument further, along the lines of what the Reviewer suggests, to point out that science is not in the business of making ontological claims, except only for practical purposes. While some of the authors of this paper are sympathetic to this view, we believe our discussion of traditional vs. fine-grained functionalism is appropriate regardless of one's views on metaphysics of science.

^2^What we mean by “approximate” here is that such systems need not necessarily meet all the criteria that characterise the organizational, living, and developmental dynamics of organismic cognition, and yet they can still exhibit interesting properties, which allows researchers to home in on the causal structure of cognition. In a sense, this is reminiscent of the role of idealisations in scientific modelling (see Potochnik, 2017; except that here such an approximating physical system would be considered both the “model” and the phenomenon model). This is not an exhaustive characterisation, partly because of what an anonymous reviewer has pointed out, namely, that some of the difficulties involved in this project stem from not having any generally accepted cases of non-biological cognition–and even cases of non-human cognition are deeply contested. However, this issue does not detract from the core arguments of the paper and so a more in-depth discussion will be left for future work.

^3^As an anonymous Reviewer pointed out, this approach is also supported by the claims made by the proponents of the Free Energy Principle framework (e.g., Friston, 2019). Exploring this topic in sufficient depth, however, would require a separate paper, and hence we have to refrain from expanding on this connection here.

^4^An interesting context here, also explored in Pfeifer's work (e.g., Pfeifer and Scheier, 1999), is the increasing popularity of “morphological computation” (see also Müller and Hoffman, 2017). For a discussion of morphological computation in the context of basal cognition (see Rorot, 2022).
